# Supplementary material for: Polygenic Scores for Schizophrenia and Educational Attainment Predict Global Functioning Across Psychiatric Hospitalization Among People with Schizophrenia
Source: medRxiv. 2025 May 21:2025.05.20.25328039. Preprint. [Version 1] doi: 10.1101/2025.05.20.25328039 (PMC12258742; doi:10.1101/2025.05.20.25328039)
Supplement: Supplement 1 [file media-1.pdf]

Supplementary Material

eMethods
-Diagnostic Procedure (p.2)
-Global Functioning (p.2)
-Global Functioning Variance Comparison (p.2)
-Deep Phenotypes (p.2)
-Phenotypic Associations (p.3)
-Genotype Quality Control (p.3)
-Linear Mixed-Effects Models (p.3)
-Autocorrelation Parameter Estimates (p.4)
-Sensitivity Analysis (p.4)
-Pseudo-R^2 Calculation and Results (p.4

eFigures
-eFigure 1. SUPER-Finland Sample Flow Diagram (p.5)
-eFigure 2. PGS Associations Stratified by Admission GF Tertile (p.6)

eTables
-eTable 1. Demographic and Descriptive Information (p.7)
-eTable 2. Estimates from Admission GF Linear Mixed-Effects Model (p.8)
-eTable 3. Estimates from Discharge GF Linear Mixed-Effects Model (p.9)
-eTable 4. Estimates from Change GF Linear Mixed-Effects Model (p.10)
-eTable 5. Multiple Comparisons Correction (p.11)
-eTable 6. Estimates from Tertile-Stratified Admission GF Linear Mixed-Effects Model (p.12)
-eTable 7. Estimates from Tertile-Stratified Discharge GF Linear Mixed-Effects Model (p.15)
-eTable 8. Estimates from Tertile-Stratified Change GF Linear Mixed-Effects Model (p.18)

eReferences

eMethods

Diagnostic Procedure

Diagnoses were based on ICD codes extracted from the Care Register for Health Care (HILMO) and the Register of Primary Health Care Visits. This approach yields highly accurate schizophrenia diagnoses.[1] Following the diagnostic procedure used in the broader SUPER-Finland study,[2] lifetime diagnoses were assigned according to a hierarchical framework in which psychotic disorders range from major depressive disorder with psychosis, to bipolar disorder, to schizoaffective disorder, to schizophrenia. Schizophrenia is at the bottom of the hierarchy. For individuals with multiple lifetime diagnoses, their final diagnosis is the lowest-hierarchy diagnosis, with schizophrenia trumping all other diagnoses. For example, individuals with a history of bipolar disorder and schizophrenia were designated as having schizophrenia. Consistent with leading genomic consortia,[3] individuals with both schizophrenia and schizoaffective disorder were included.

Global Functioning

Since 1994, global functioning has been collected as a routine part of psychiatric inpatient admission and discharge in Finland.[4] Global Assessment Scale (GAS) scores[5] are assigned by clinicians who are all held to the same standards of practice and who undergo similar psychiatric training. The same GAS rating guidelines are followed nationwide.[4] GAS scores range from 1 to 100, with higher scores indicating better functioning. The GAS is analogous to other widely used functional measures including the Global Assessment of Functioning (GAF). Its reliability is acceptable,[5] including in clinical settings where GAS scores are collected longitudinally by multiple raters,[6] and sufficient for analyses of group-level change in functioning over time.[7]

Because GF scores were not collected systematically until January 1, 1994, we excluded a small number of sporadic GF scores collected prior to that date. We observed low rates of missingness in the remaining scores, which can be considered missing at random. 7.37% and 5.03% of post-1993 psychiatric admission and discharge GF scores were missing, respectively. The discrepancy in missingness rates (i.e., higher rate of Admission GF missingness) was driven by hospitalizations where admission occurred in 1993 (immediately prior to the start of systematic GF collection) and discharge took place after the turn of the new year.

In situations where hospitalizations were immediately adjacent (e.g., discharge followed by admission on the same day), we merged hospitalizations and extracted the first admission GF score and the last discharge GF score. In addition to the 1-100 score range, the GAS allows clinicians to assign a zero if they did not have enough information to assess a patient’s global functioning. We recoded zeroes to NA prior to analysis.

For SUPER-Finland participants who met our inclusion criteria, a total of 117,810 admission and discharge GAS scores were available. The most recent score available was collected in February 2019.

Global Functioning Variance Comparison

To compare the variances of Admission GF and Discharge GF (SD = 12.31 and 11.85, respectively), we performed a Brown-Forsythe test.[8] The test was significant ($F(1, 117{,}808) = 378.97$, $P < .001$), indicating that phenotypic variance in Admission GF was significantly larger than Discharge GF variance. This result suggests that variance decreased as patients stabilized and clinicians gathered additional information about their functional status

Deep Phenotypes

We examined associations between GF scores and a variety of phenotypes available in the SUPER-Finland study, prioritizing clinically relevant outcomes.

From the Care Register for Health Care, HILMO, we calculated psychiatric hospitalization burden including number of hospitalizations, total days in the hospital, and median length of stay. We focused on psychiatric hospitalizations during the five years following participants’ first hospitalization because nearly all the sample had at least five years of follow-up data available.

SUPER-Finland participants completed an interview that was based on questions from the Finnish Health 2000 and 2011 general population surveys.[9,10] We analyzed educational attainment from the interview and in a small number of cases where it was unavailable, it was extracted from the Register for Completed Education and Degrees. Following the population surveys, educational attainment was coded as one of three levels: basic (compulsory education; no completion of vocational school or the matriculation examination), secondary (passed matriculation

examination but no degree; completion of vocational school), and higher (degree from university, polytechnic institute, or higher vocational institution). History of difficulties at school that lasted for more than a year was also collected during the interview. Suicide attempt history was collected using a questionnaire.

Cognitive ability was assessed at entry to the SUPER-Finland study (i.e., between November 2015 and December 2018) using two, Finnish-adapted subtests of the Cambridge Neuropsychological Test Automated Battery (CANTAB): Paired Associates Learning (PAL), which measures visual memory, and Reaction Time (RTI).[11] We analyzed only CANTAB data that were deemed "Complete and Reliable” by research supervisors. For the RTI, which was the five-choice version, better performance is indicated by quicker median reaction time and fewer errors. Better performance on the PAL is indicated by more patterns reached, more total attempts, higher first attempt memory, and fewer total errors. We used an adjusted total errors metric that estimated the number of errors a participant would have made had they completed all trials.

Phenotypic Associations

To examine the phenotypic correlates of our GF scores, we performed an exploratory analysis investigating their associations with the deep phenotypes listed above. Specifically, we fit a series of linear mixed-effects models in which each deep phenotype was modeled as a predictor of each GF score. Analogously to our genetic analyses, hospitalizations were nested within individual participants using a random intercept. All analyses controlled for sex and year of birth.

Genotype Quality Control and Imputation

Samples were genotyped in four batches at the Broad Institute of MIT and Harvard using the Illumina Global Screening Array (version 1). Within-batch QC excluded samples with poor genotype quality, genetically inferred sex mismatching self-reported sex, sample missingness > 0.05, and Hardy-Weinberg equilibrium (HWE) $P < 1 \times 10^{-12}$. Batches were then merged before undergoing another round of QC, which applied the same filters except the HWE and missingness thresholds were set at $P < 1 \times 10^{-9}$ and > 0.02, respectively. We excluded variants with minor allele frequency (MAF) < 0.01. PLINK 2[12] was used to calculate genetic principal components. Seventy-one participants with non-European ancestry as inferred from the principal components were excluded, as this sample size was insufficient for robust genetic analyses. Imputation was performed using Sequencing Initiative Suomi (SiSu) v.3, a Finnish-specific reference panel.[13] Variants with INFO score < 0.8 were excluded.

Linear Mixed-Effects Models

We fit a series of linear mixed-effects models using the nlme R package.[14,15] Separate models were fit to global functioning scores at psychiatric admission and discharge, as well as functional change in response to hospitalization (Change GF; Discharge GF adjusted for Admission GF). For hospitalization i and individual j:

$$\mathrm{GF}_{ij} = \beta_0 + \beta_1 \mathrm{SZ}_j + \beta_2 \mathrm{EA}_j + \beta_3 \mathrm{Sex}_j + \beta_4 \mathrm{YOB}_j + \mathbf{X}_j \mathbf{b}_X + u_{0j} + \varepsilon_{ij} \quad (1)$$

We were primarily interested in the fixed effects of schizophrenia (SZ) and educational attainment (EA) polygenic scores. Sex (coded as Female/Male), year of birth (YOB), and the first 10 genetic principal components are included as fixed covariates. X is a design matrix of the principal components and b is a vector containing their regression coefficients. Discharge GF and functional change analyses also controlled for the log of length of stay at hospitalization i (not shown), and the analysis of functional change in response to hospitalization controlled for Admission GF (not shown). To account for the repeated GF measures, hospitalization is nested within participant j using random intercept $u0_j$. Autocorrelation in residuals ϵ_{ij} of GF across hospitalizations is handled by specifying a first-order autocorrelation structure in continuous time, which is robust to unequal temporal spacing of observations.[16] The correlation between residuals at any pair of hospitalizations i and k for individual j is modeled as:

$$\mathrm{Cor}(\varepsilon_{ij}, \varepsilon_{kj}) = \rho^{|t_{ij} - t_{kj}|} \quad (2)$$

Autocorrelation parameter ρ decays exponentially with the time elapsed between discharge dates t_{ij} and t_{kj} such that residual correlations are stronger among temporally closer GF measures and weaker among more distal hospitalizations.

Autocorrelation Parameter Estimates
Results from our linear mixed-effects model analyses are discussed in the main manuscript. Parameter estimates are presented in eTables 2-4.

Autocorrelation parameter estimates (ρ) equaled 0.93, 0.96, and 0.93 in the Admission GF, Discharge GF, and Change GF analyses, respectively. These estimates indicate strong positive autocorrelation in the residuals of the repeated GF measures, which we modeled as a first-order residual autocorrelation structure in continuous time.

Sensitivity Analysis
To confirm that PGS associations were consistent across GF strata, we performed an exploratory, post-hoc sensitivity analysis in which we separated hospitalizations based on Admission GF tertile and re-ran our linear mixed-effects models within each tertile. Lower, Middle, and Upper tertiles corresponded to Admission GF scores $\leq$ 32, between 33 and 45 (inclusive), and $\geq$ 46, respectively. Results are presented in eFigure 2 and eTables 6-8. SZ PGS associations with Discharge GF and functional change were significantly negative across all tertiles (*P*s < .05), consistent with results from our main analysis. As in the main analysis, EA PGS was positively associated with Discharge GF and functional change across all tertiles. However, none of these associations were significant (*P*s > .05), unlike the main analysis in which the functional change association was significant. This attenuation of significance is likely driven by power reductions due to analyzing only one third of available hospitalizations. No Admission GF associations were significant (*P*s > .05), reflecting the power reduction and the decreased Admission GF variability as a result of conditioning the sensitivity analysis on Admission GF tertile.

Pseudo-R^2 Calculation and Results
To estimate the proportion of variance explained by our models, we calculated pseudo-R^2 values[17] and the MuMIn package.[18] This approach yields two estimates: marginal R^2, which estimates the proportion of variation in GF explained by the fixed effects alone, and conditional R^2, which estimates the proportion of variance explained by both fixed and random effects. Conditional R^2 for the Admission GF, Discharge GF, and functional change analyses equaled 0.02, 0.02, and 0.24, respectively. Marginal R^2 equaled 0.27, 0.26, and 0.43, respectively (eTables 2-4). Our observation of larger pseudo-R^2 values for the functional change analysis was driven by the inclusion of Admission GF in the model.

eFigures

eFigure 1. SUPER-Finland Sample Flow Diagram

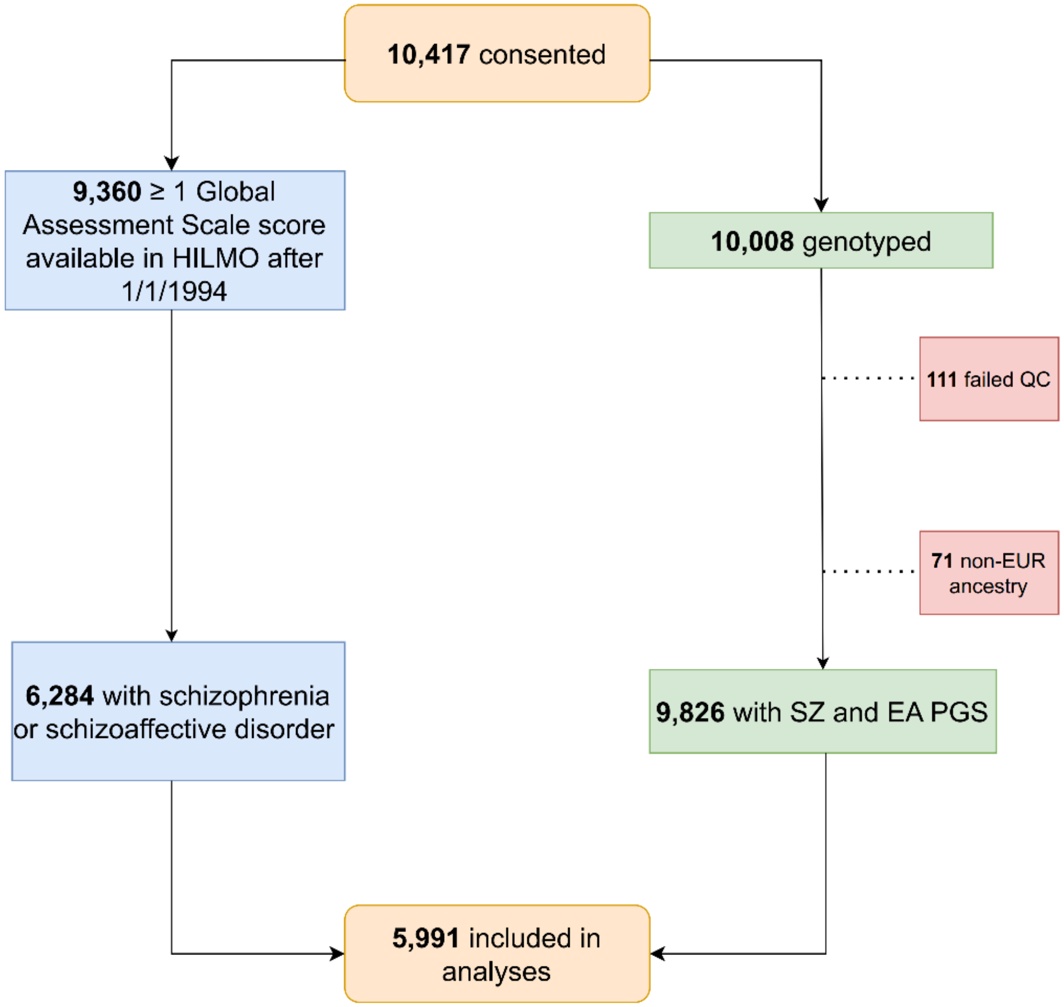

HILMO: Care Register for Health Care. SZ: schizophrenia. EA: educational attainment. PGS: polygenic score. EUR: European. QC: quality control.

eFigure 2. PGS Associations Stratified by Admission GF Tertile

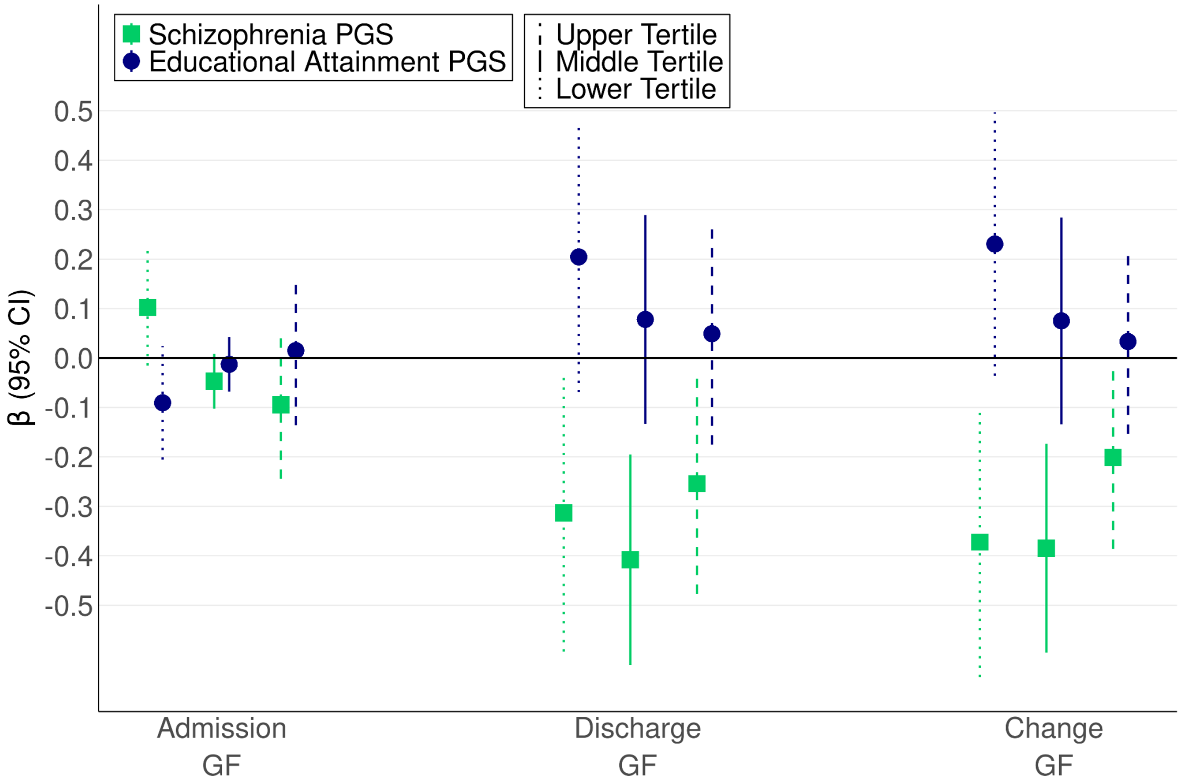

PGS: polygenic score. GF: global functioning. Change GF: Discharge GF adjusted for Admission GF. Bars depict 95% CIs.

eTables

eTable 1. Demographic and Descriptive Information

Domain	Phenotype	n (%)	Median	IQR
Demographics	Sex (Female/Male)	2733 (45.62) / 3258 (54.38)	-	-
	Age (Years)	5991 (100)	47	20
	Diagnosis (Schizophrenia / Schizoaffective)	5155 (86.05) / 836 (13.95)	-	-
Clinical Status and Inpatient Psychiatric History	Hospitalizations with GF (Post-December 31, 1993)	5991 (100)	6	9
	Admission GF*	5947 (99.27)	39	16
	Discharge GF*	5991 (100)	50	16
	Length of Psychiatric Stay (Days)*	5991 (100)	14	38
	Psychiatric Admissions First 5 years	5991 (100)	3	4
	Days in Psychiatric Hospital First 5 years	5991(100)	158	290
	Suicide Attempt History (Yes / No)	3390 (56.58) / 2435 (40.64)	-	-
Education	Educational Attainment (Basic / Secondary / Higher)	2238 (37.36) / 2681 (44.75) / 1018 (16.99)	-	-
	School Difficulties History (Yes / No)	2837 (47.35) / 2990 (49.91)	-	-
CANTAB	RTI Median Reaction Time (ms)	3921 (65.45)	436	88
	RTI All Errors	3921 (65.45)	0	1
	PAL Patterns Reached	3732 (62.29)	6	4
	PAL Total Attempts	3732 (62.29)	7	3
	PAL First Attempt Memory	3732 (62.29)	7	7
	PAL Total Errors Adjusted	3732 (62.29)	40	37

IQR: interquartile range. GF: global functioning. CANTAB: Cambridge Neuropsychological Test Automated Battery. RTI: Reaction Time. PAL: Paired Associates Learning. * To account for repeated measures when estimating descriptive statistics for these variables, we performed cluster bootstrapping by resampling at the individual participant level (1000 iterations).[19]

eTable 2. Estimates from Admission GF Linear Mixed-Effects Model

Fixed Effects					
	Estimate/β	SE	95% CI	*t*	*P*
Intercept	163.62	14.38	135.43 to 191.81	11.38	< .001
SZ PGS	−0.20	0.10	-0.40 to -7.28×10^{-6}	−1.96	.050
EA PGS	−0.23	0.10	−0.43 to −0.04	−2.32	.020
Sex	−0.13	0.20	−0.52 to 0.26	−0.67	.502
Year of Birth	−0.06	0.01	−0.08 to −0.05	−8.82	< .001
PC 1	62.93	14.31	34.87 to 91.00	4.40	< .001
PC 2	−115.28	13.22	−141.21 to −89.36	−8.72	< .001
PC 3	108.66	13.34	82.50 to 134.81	8.14	< .001
PC 4	−4.03	14.53	−32.51 to 24.45	−0.28	.782
PC 5	−56.03	13.18	−81.87 to −30.19	−4.25	< .001
PC 6	27.86	13.17	2.04 to 53.68	2.12	.035
PC 7	73.40	13.99	45.98 to 100.83	5.25	< .001
PC 8	−25.88	13.37	−52.10 to 0.33	−1.94	.053
PC 9	8.71	10.06	−11.01 to 28.43	0.87	.039
PC 10	67.94	12.67	43.08 to 92.80	5.36	< .001
Random Effect					
				Variance	SD
Participant (Intercept)				36.12	6.01
Goodness of Fit					
				Marginal	Conditional
Pseudo-R^2				0.02	0.27

SZ: schizophrenia. EA: educational attainment. PGS: polygenic score. PC: principal component. Sex was coded as a binary variable (Female/Male).

eTable 3. Estimates from Discharge GF Linear Mixed-Effects Model

Fixed Effects					
	Estimate/β	SE	95% CI	*t*	*P*
Intercept	1.78	14.26	−26.16 to 29.73	0.13	.901
SZ PGS	−0.36	0.10	−0.56 to −0.17	−3.60	< .001
EA PGS	0.15	0.10	−0.04 to 0.35	1.56	.119
Sex	−0.67	0.20	−1.05 to -0.28	−3.37	< .001
Year of Birth	0.03	0.01	0.01 to 0.04	3.51	< .001
Length of Stay	−0.13	0.03	−0.20 to −0.07	−3.98	< .001
PC 1	98.10	14.15	70.36 to 125.85	6.93	< .001
PC 2	−74.00	13.14	−99.76 to −48.23	−5.63	< .001
PC 3	35.98	13.25	10.01 to 61.95	2.72	.007
PC 4	31.60	14.46	3.25 to 59.94	2.19	.029
PC 5	−46.74	13.07	−72.37 to −21.12	−3.58	< .001
PC 6	24.62	13.08	−1.03 to 50.26	1.88	.060
PC 7	62.32	13.85	35.16 to 89.48	4.50	< .001
PC 8	9.93	13.22	−15.99 to 35.84	0.75	.453
PC 9	16.31	9.97	−3.23 to 35.86	1.64	.102
PC 10	80.99	12.59	56.31 to 105.68	6.43	< .001
Random Effect					
				Variance	SD
Participant (Intercept)				35.70	5.97
Goodness of Fit					
				Marginal	Conditional
Pseudo-R^2				0.02	0.26

SZ: schizophrenia. EA: educational attainment. PGS: polygenic score. PC: principal component. Sex was coded as a binary variable (Female/Male). Length of stay was scaled in days and log-transformed.

eTable 4. Estimates from Change GF Linear Mixed-Effects Model

Fixed Effects					
	Estimate/β	SE	95% CI	*t*	*P*
Intercept	−63.44	12.49	−87.93 to −38.95	−5.08	< .001
SZ PGS	−0.31	0.09	−0.49 to −0.14	−3.56	< .001
EA PGS	0.20	0.09	0.03 to 0.37	2.27	.023
Admission GF	0.48	0.004	0.47 to 0.49	129.18	< .001
Sex	−0.62	0.17	−0.96 to −0.28	−3.60	< .001
Year of Birth	0.05	0.006	0.04 to 0.06	7.52	< .001
Length of Stay	1.05	0.03	0.99 to 1.12	33.16	< .001
PC 1	68.28	12.43	43.91 to 92.65	5.49	< .001
PC 2	−24.57	11.49	−47.09 to −2.05	−2.14	.033
PC 3	−12.52	11.59	−35.24 to 10.20	−1.08	.280
PC 4	28.03	12.61	3.30 to 52.75	2.22	.026
PC 5	−22.77	11.44	−45.21 to −0.34	−1.99	.047
PC 6	9.57	11.44	−12.84 to 31.99	0.84	.403
PC 7	28.68	12.15	4.86 to 52.50	2.36	.018
PC 8	19.87	11.60	−2.88 to 42.62	1.71	.087
PC 9	10.81	8.73	−6.31 to 27.93	1.24	.216
PC 10	50.02	11.01	28.43 to 71.62	4.54	< .001
Random Effect					
				Variance	SD
Participant (Intercept)				27.65	5.26
Goodness of Fit					
				Marginal	Conditional
Pseudo-R^2				0.24	0.43

Change GF: Discharge GF adjusted for Admission GF. SZ: schizophrenia. EA: educational attainment. PGS: polygenic score. PC: principal component. Sex was coded as a binary variable (Female/Male). Length of stay was scaled in days and log-transformed.

eTable 5. Multiple Comparisons Correction

Fixed Effects			
Predictor	Outcome	Unadjusted *P*	FDR-Adjusted *P*
SZ PGS	Admission GF	.0500	.0600
	Discharge GF*	.0003	.0012
	Change GF*	.0004	.0012
EA PGS	Admission GF*	.0204	.0348
	Discharge GF	.1191	.1191
	Change GF*	.0232	.0348

SZ: schizophrenia. EA: educational attainment. PGS: polygenic score. GF: global functioning. Change GF: discharge GF adjusted for admission GF. FDR: false discovery rate, which we controlled using the Benjamini-Hochberg procedure.[20] *Significant at .05 after FDR adjustment.

eTable 6. Estimates from Tertile-Stratified Admission GF Linear Mixed-Effects Model

Fixed Effects						
	Tertile	Estimate/β	SE	95% CI	*t*	*P*
Intercept	Upper	107.74	11.63	84.95 to 130.53	9.27	< .001
	Middle	45.03	4.13	36.93 to 53.12	10.90	< .001
	Lower	45.97	8.44	29.42 to 62.51	5.45	< .001
SZ PGS	Upper	0.09	0.08	−0.24 to 0.05	−1.24	.214
	Middle	−0.05	0.03	−0.10 to 0.01	-0.45	.097
	Lower	0.10	0.06	−0.02 to 0.22	1.70	.088
EA PGS	Upper	0.02	0.08	−0.14 to 0.17	0.20	.845
	Middle	−0.01	0.03	−0.07 to 0.04	−0.45	.650
	Lower	−0.09	0.06	−0.21 to 0.02	−1.55	.122
Sex	Upper	0.08	0.15	−0.22 to 0.38	0.53	.596
	Middle	−0.10	0.06	−0.20 to 0.01	−1.72	.085
	Lower	−0.30	0.12	−0.53 to −0.07	−2.57	.010
Year of Birth	Upper	−0.03	0.01	−0.04 to −0.02	−4.82	< .001
	Middle	−0.003	0.002	−0.01 to 0.001	−1.66	.098
	Lower	−0.01	0.004	−0.02 to −0.002	−2.45	.015
PC 1	Upper	32.36	11.11	10.58 to 54.14	2.91	.004
	Middle	14.29	4.05	6.34 to 22.24	3.52	< .001
	Lower	18.42	8.40	1.95 to 34.90	2.19	.028
PC 2	Upper	0.002	10.23	−20.05 to 20.06	1.65×10^{-4}	1.00
	Middle	−1.18	3.81	−8.65 to 6.30	−0.31	.758
	Lower	−22.12	7.91	−37.63 to −6.61	−2.80	.005
PC 3	Upper	13.50	10.69	−7.46 to 34.46	1.26	.207
	Middle	19.85	3.87	12.25 to 27.44	5.12	< .001
	Lower	6.15	7.80	−9.14 to 21.44	0.79	.431
PC 4	Upper	21.76	10.88	0.43 to 43.10	2.00	.046
	Middle	−8.50	4.35	−17.03 to 0.02	−1.96	.051

	Tertile	Estimate/β	SE	95% CI	*t*	*P*
	Lower	8.38	8.84	−8.95 to 25.72	0.95	.343
PC 5	Upper	1.23	10.08	−18.53 to 20.98	0.12	.903
	Middle	4.16	3.74	−3.17 to 11.49	1.11	.266
	Lower	0.53	7.85	−14.87 to 15.93	0.07	.946
PC 6	Upper	21.64	9.58	2.86 to 40.42	2.26	.024
	Middle	1.73	3.82	−5.75 to 9.21	0.45	.650
	Lower	15.96	8.07	0.14 to 31.78	1.98	.048
PC 7	Upper	−6.64	10.67	−27.56 to 14.27	−0.62	.534
	Middle	8.94	3.94	1.22 to 16.67	2.27	.023
	Lower	27.06	8.29	10.82 to 43.31	3.27	.001
PC 8	Upper	9.46	10.53	−11.18 to 30.10	0.90	.369
	Middle	−4.69	3.77	−12.09 to 2.70	−1.24	.214
	Lower	7.35	7.89	−8.12 to 22.82	0.93	.352
PC 9	Upper	−9.29	8.09	−25.16 to 6.58	−1.15	.251
	Middle	3.20	2.90	−2.48 to 8.88	1.10	.270
	Lower	3.73	5.39	−6.84 to 14.29	0.69	.489
PC 10	Upper	5.51	9.12	−12.38 to 23.39	0.60	.546
	Middle	4.30	3.54	−2.65 to 11.25	1.21	.225
	Lower	16.13	7.75	0.94 to 31.31	2.08	.037
Random Effect						
					Variance	SD
Participant (Intercept)	Upper				7.96	2.82
	Middle				0.93	0.96
	Lower				5.18	2.28
Goodness of Fit						
					Marginal	Conditional
Pseudo-R^2	Upper				0.01	0.19

			Marginal	Conditional
	Middle		0.01	0.11
	Lower		0.004	0.13

SZ: schizophrenia. EA: educational attainment. PGS: polygenic score. PC: principal component. Sex was coded as a binary variable (Female/Male).

eTable 7. Estimates from Tertile-Stratified Discharge GF Linear Mixed-Effects Model

Fixed Effects						
	Tertile	Estimate/**β**	SE	95% CI	*t*	*P*
Intercept	Upper	48.02	17.11	14.48 to 81.55	2.81	.005
	Middle	−26.02	15.63	−56.66 to 4.61	−1.66	.096
	Lower	−77.29	20.02	−116.53 to −38.05	−3.86	< .001
SZ PGS	Upper	−0.25	0.11	−0.48 to −0.03	−2.24	.025
	Middle	−0.41	0.11	−0.62 to −0.19	−3.75	< .001
	Lower	−0.31	0.14	−0.59 to −0.03	−2.19	.028
EA PGS	Upper	0.05	0.11	−0.18 to 0.27	0.43	.667
	Middle	0.08	0.11	−0.13 to 0.29	0.72	.469
	Lower	0.20	0.14	−0.07 to 0.48	1.46	.143
Sex	Upper	−0.37	0.23	−0.81 to 0.07	−1.63	.103
	Middle	−0.63	0.21	−1.04 to −0.21	−2.94	.003
	Lower	−0.86	0.28	−1.40 to −0.31	−3.08	.002
Year of Birth	Upper	0.00	0.01	−0.01 to 0.02	0.54	.586
	Middle	0.04	0.01	0.02 to 0.05	4.73	< .001
	Lower	0.06	0.01	0.04 to 0.08	6.06	< .001
Length of Stay	Upper	0.21	0.05	0.11 to 0.31	4.10	< .001
	Middle	0.99	0.05	0.89 to 1.09	19.82	< .001
	Lower	0.89	0.07	0.76 to 1.02	13.33	< .001
PC 1	Upper	65.98	16.48	33.67 to 98.30	4.00	< .001
	Middle	58.94	15.48	28.60 to 89.29	3.81	< .001
	Lower	95.05	20.00	55.84 to 134.27	4.75	< .001
PC 2	Upper	4.61	15.05	−24.89 to 34.12	0.31	.759
	Middle	−15.89	14.49	−44.30 to 12.51	−1.10	.273
	Lower	−55.31	18.74	−92.05 to −18.57	−2.95	.003
PC 3	Upper	13.70	15.65	−16.98 to 44.39	0.88	.381

	Tertile	Estimate/β	SE	95% CI	*t*	*P*
	Middle	−6.10	14.75	−35.02 to 22.82	−0.41	.679
	Lower	−18.63	18.52	−54.93 to 17.67	−1.01	.314
PC 4	Upper	73.95	16.11	42.37 to 105.53	4.59	< .001
	Middle	18.36	16.31	−13.61 to 50.33	1.13	.260
	Lower	14.54	20.77	−26.18 to 55.27	0.70	.484
PC 5	Upper	6.68	14.89	−22.52 to 35.88	0.45	.654
	Middle	−20.29	14.32	−48.37 to 7.79	−1.42	.157
	Lower	−32.34	18.64	−68.89 to 4.21	−1.73	.083
PC 6	Upper	23.20	14.19	−4.63 to 51.02	1.63	.102
	Middle	−4.57	14.52	−33.02 to 23.89	−0.31	.753
	Lower	32.86	19.07	−4.52 to 70.24	1.72	.085
PC 7	Upper	−11.00	15.84	−42.06 to 20.06	−0.69	.488
	Middle	38.67	15.15	8.96 to 68.38	2.55	.011
	Lower	60.20	19.69	21.60 to 98.79	3.06	.002
PC 8	Upper	11.42	15.55	−19.08 to 41.92	0.73	.463
	Middle	4.98	14.40	-23.25 to 33.21	0.35	.730
	Lower	37.09	18.77	0.29 to 73.88	1.98	.048
PC 9	Upper	−7.00	11.78	−30.10 to 16.10	−0.59	.553
	Middle	22.72	11.19	0.77 to 44.67	2.03	.043
	Lower	16.20	13.00	−9.28 to 41.69	1.25	.213
PC 10	Upper	22.93	13.60	−3.72 to 49.59	1.69	.092
	Middle	63.89	13.56	37.31 to 90.46	4.71	< .001
	Lower	65.73	18.34	29.77 to 101.68	3.58	< .001
Random Effect						
					Variance	SD
Participant (Intercept)	Upper				23.44	4.84
	Middle				26.48	5.15

			Variance	SD
	Lower		46.43	6.81
Goodness of Fit				
			Marginal	Conditional
Pseudo-R^2	Upper		0.01	0.30
	Middle		0.03	0.31
	Lower		0.02	0.28

SZ: schizophrenia. EA: educational attainment. PGS: polygenic score. PC: principal component. Sex was coded as a binary variable (Female/Male). Length of stay was scaled in days and log-transformed.

eTable 8. Estimates from Tertile-Stratified Change GF Linear Mixed-Effects Model

Fixed Effects						
	Tertile	Estimate/β	SE	95% CI	*t*	*P*
Intercept	Upper	-25.58	14.24	-53.49 to 2.33	-1.80	.073
	Middle	-47.47	15.51	-77.87 to -17.07	-3.06	.002
	Lower	-97.62	19.50	-135.84 to -59.41	-5.01	< .001
SZ PGS	Upper	-0.20	0.09	-0.39 to -0.02	-2.12	.034
	Middle	-0.38	0.11	-0.60 to -0.17	-3.57	< .001
	Lower	-0.37	0.14	-0.65 to -0.10	-2.68	.008
EA PGS	Upper	0.03	0.10	-0.15 to 0.22	0.35	.726
	Middle	0.08	0.11	-0.13 to 0.28	0.70	.481
	Lower	0.23	0.14	-0.04 to 0.50	1.70	.090
Admission GF	Upper	0.73	0.01	0.71 to 0.74	95.33	< .001
	Middle	0.48	0.02	0.44 to 0.52	22.70	< .001
	Lower	0.44	0.01	0.41 to 0.47	31.36	< .001
Sex	Upper	-0.42	0.19	-0.79 to -0.05	-2.24	.025
	Middle	-0.57	0.21	-0.98 to -0.16	-2.70	.007
	Lower	-0.72	0.27	-1.25 to -0.18	-2.64	.008
Year of Birth	Upper	0.02	0.01	0.01 to 0.04	3.09	.002
	Middle	0.04	0.01	0.02 to 0.05	4.96	< .001
	Lower	0.07	0.01	0.05 to 0.09	6.66	< .001
Length of Stay	Upper	0.77	0.04	0.69 to 0.86	18.10	< .001
	Middle	1.11	0.05	1.02 to 1.21	22.47	< .001
	Lower	1.08	0.07	0.96 to 1.21	16.63	< .001
PC 1	Upper	44.08	13.71	17.19 to 70.97	3.21	.001
	Middle	51.63	15.33	21.57 to 81.69	3.37	< .001
	Lower	86.04	19.47	47.87 to 124.21	4.42	< .001
PC 2	Upper	5.50	12.52	-19.05 to 30.05	0.44	.661
	Middle	-14.22	14.35	-42.36 to 13.92	-0.99	.322

	Tertile	Estimate/β	SE	95% CI	*t*	*P*
	Lower	-46.87	18.24	-82.63 to -11.11	-2.57	.010
PC 3	Upper	2.91	13.01	-22.61 to 28.42	0.22	.823
	Middle	-15.41	14.62	-44.08 to 13.25	-1.05	.292
	Lower	-21.25	18.02	-56.58 to 14.07	-1.18	.238
PC 4	Upper	55.74	13.42	29.43 to 82.05	4.15	< .001
	Middle	21.77	16.15	-9.89 to 53.43	1.35	.178
	Lower	9.59	20.21	-30.04 to 49.22	0.47	.635
PC 5	Upper	6.90	12.39	-17.40 to 31.20	0.56	.578
	Middle	-21.30	14.19	-49.12 to 6.52	-1.50	.133
	Lower	-31.94	18.15	-67.52 to 3.63	-1.76	.078
PC 6	Upper	8.28	11.82	-14.90 to 31.46	0.70	.484
	Middle	-6.10	14.38	-34.29 to 22.09	-0.42	.671
	Lower	25.03	18.56	-11.35 to 61.41	1.35	.178
PC 7	Upper	-7.14	13.19	-33.00 to 18.72	-0.54	.588
	Middle	34.58	15.01	5.14 to 64.01	2.30	.021
	Lower	48.93	19.16	11.35 to 86.50	2.55	.011
PC 8	Upper	4.28	12.94	-21.09 to 29.65	0.33	.741
	Middle	7.10	14.26	-20.87 to 35.06	0.50	.619
	Lower	33.88	18.27	-1.93 to 69.69	1.85	.064
PC 9	Upper	-0.59	9.80	-19.79 to 18.62	-0.06	.952
	Middle	21.47	11.09	-0.28 to 43.22	1.94	.053
	Lower	14.52	12.65	-10.29 to 39.32	1.15	.251
PC 10	Upper	18.71	11.33	-3.51 to 40.92	1.65	.099
	Middle	62.15	13.43	35.83 to 88.48	4.63	< .001
	Lower	58.15	17.85	23.16 to 93.14	3.26	.001

Random Effect				
			Variance	SD
Participant (Intercept)	Upper		17.31	4.16
	Middle		26.27	5.13
	Lower		44.00	6.63
Goodness of Fit				
			Marginal	Conditional
Pseudo-R^2	Upper		0.30	0.53
	Middle		0.05	0.33
	Lower		0.07	0.31

Change GF: Discharge GF adjusted for Admission GF. SZ: schizophrenia. EA: educational attainment. PGS: polygenic score. PC: principal component. Sex was coded as a binary variable (Female/Male). Length of stay was scaled in days and log-transformed.

eReferences

1. Pihlajamaa J, Suvisaari J, Henriksson M, et al. The validity of schizophrenia diagnosis in the Finnish Hospital Discharge Register: Findings from a 10-year birth cohort sample. *Nord J Psychiatry*. 2008;62(3):198-203. doi:10.1080/08039480801983596
2. Lähteenvuo M, Ahola-Olli A, Suokas K, et al. Cohort profile: SUPER-Finland – the Finnish study for hereditary mechanisms of psychotic disorders. *BMJ Open*. 2023;13(4):e070710. doi:10.1136/BMJOPEN-2022-070710
3. Trubetskoy V, Pardiñas AF, Qi T, et al. Mapping genomic loci implicates genes and synaptic biology in schizophrenia. *Nat 2022 6047906*. 2022;604(7906):502-508. doi:10.1038/s41586-022-04434-5
4. Hartzell J, Järvelin J, Kyrölä A, et al. *Hoitoilmoitusopas Terveydenhuollolle Ja Kotihoidolle 2025*.; 2025.
5. Endicott J, Spitzer RL, Fleiss JL, Cohen J. The Global Assessment Scale: A Procedure for Measuring Overall Severity of Psychiatric Disturbance. *Arch Gen Psychiatry*. 1976;33(6):766-771. doi:10.1001/ARCHPSYC.1976.01770060086012
6. Dworkin RJ, Friedman LC, Telschow RL, Grant KD, Moffic HS, Sloan VJ. The longitudinal use of the Global Assessment Scale in multiple-rater situations. *Community Ment Health J*. 1990;26(4):335-344. doi:10.1007/BF00752724/METRICS
7. Söderberg P, Tungström S, Armelius BÅ. Reliability of global assessment of functioning ratings made by clinical psychiatric staff. *Psychiatr Serv*. 2005;56(4):434-438. doi:10.1176/APPI.PS.56.4.434/ASSET/IMAGES/IY12T4.JPEG
8. Brown MB, Forsythe AB. Robust tests for the equality of variances. *J Am Stat Assoc*. 1974;69(346):364-367. doi:10.1080/01621459.1974.10482955
9. Aromaa A, Koskinen S. *Health and Functional Capacity in Finland. Baseline Results of the Health 2000 Health Examination Survey.*; 2004.
10. Latvala A, Tuulio-Henriksson A, Perälä J, et al. Prevalence and correlates of alcohol and other substance use disorders in young adulthood: A population-based study. *BMC Psychiatry*. 2009;9(1):1-14. doi:10.1186/1471-244X-9-73/TABLES/4
11. Sahakian BJ, Morris RG, Evenden JL, et al. A comparative study of visuospatial memory and learning in Alzheimer-type dementia and Parkinson's Disease. *Brain*. 1988;111(3):695-718. doi:10.1093/BRAIN/111.3.695
12. Chang CC, Chow CC, Tellier LCAM, Vattikuti S, Purcell SM, Lee JJ. Second-generation PLINK: Rising to the challenge of larger and richer datasets. *GigaScience*. 2015;4(1):7. doi:10.1186/S13742-015-0047-8/2707533
13. Kurki MI, Karjalainen J, Palta P, et al. FinnGen provides genetic insights from a well-phenotyped isolated population. *Nat 2023 6137944*. 2023;613(7944):508-518. doi:10.1038/s41586-022-05473-8
14. Pinheiro J, Bates D, R Core Team. Linear and Nonlinear Mixed Effects Models. *CRAN Contrib Packag*. Published online January 27, 2025. doi:10.32614/CRAN.PACKAGE.NLME
15. R Core Team. R: A Language and Environment for Statistical Computing. Published online 2025.
16. Singer JD, Willett JB. *Applied Longitudinal Data Analysis: Modeling Change and Event Occurrence*. Oxford University Press; 2003.
17. Nakagawa S, Schielzeth H. A general and simple method for obtaining R2 from generalized linear mixed-effects models. *Methods Ecol Evol*. 2013;4(2):133-142. doi:10.1111/J.2041-210X.2012.00261.X
18. Bartoń K. MuMIn: Multi-Model Inference. *CRAN Contrib Packag*. Published online 2025.
19. Canty A, Ripley BD. boot: Bootstrap R (S-Plus) Functions. *Bootstrap Methods Their Appl*. Published online 2024. doi:10.1017/CBO9780511802843
20. Benjamini Y, Hochberg Y. Controlling the False Discovery Rate: A Practical and Powerful Approach to Multiple Testing. *J R Stat Soc Ser B Stat Methodol*. 1995;57(1):289-300. doi:10.1111/J.2517-6161.1995.TB02031.X
