# Supplementary material for: Polygenic Scores for Schizophrenia and Educational Attainment Predict Global Functioning Across Psychiatric Hospitalization Among People with Schizophrenia
Source: medRxiv. 2025 May 21:2025.05.20.25328039. Preprint. [Version 1] doi: 10.1101/2025.05.20.25328039 (PMC12258742; doi:10.1101/2025.05.20.25328039)
Supplement: Supplement 2 [file media-2.pdf]

Figure 1. Global Functioning Across 59,795 Psychiatric Hospitalizations

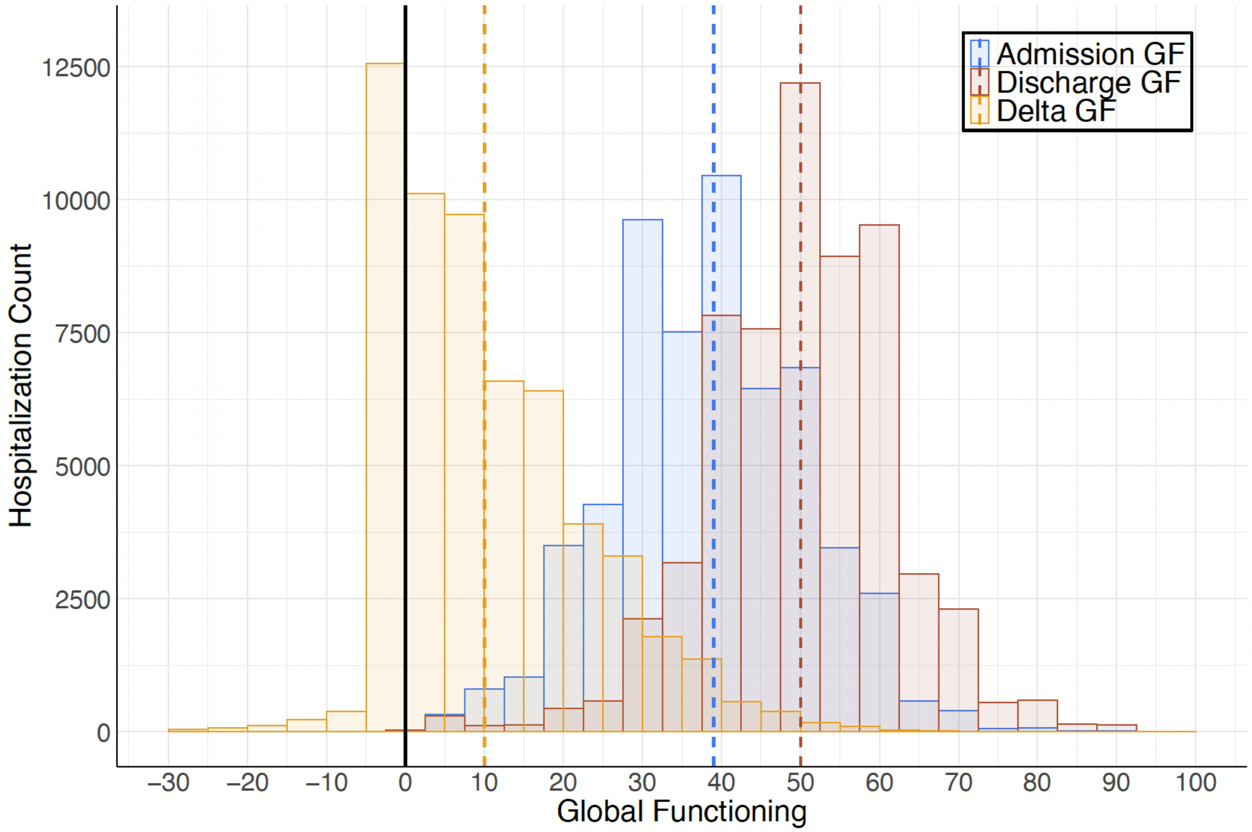

GF: global functioning. Delta GF = Discharge GF - Admission GF. Dashed vertical lines depict median GF scores.

Figure 2. Phenotypic Associations Among Global Functioning Scores and Deep Phenotypes

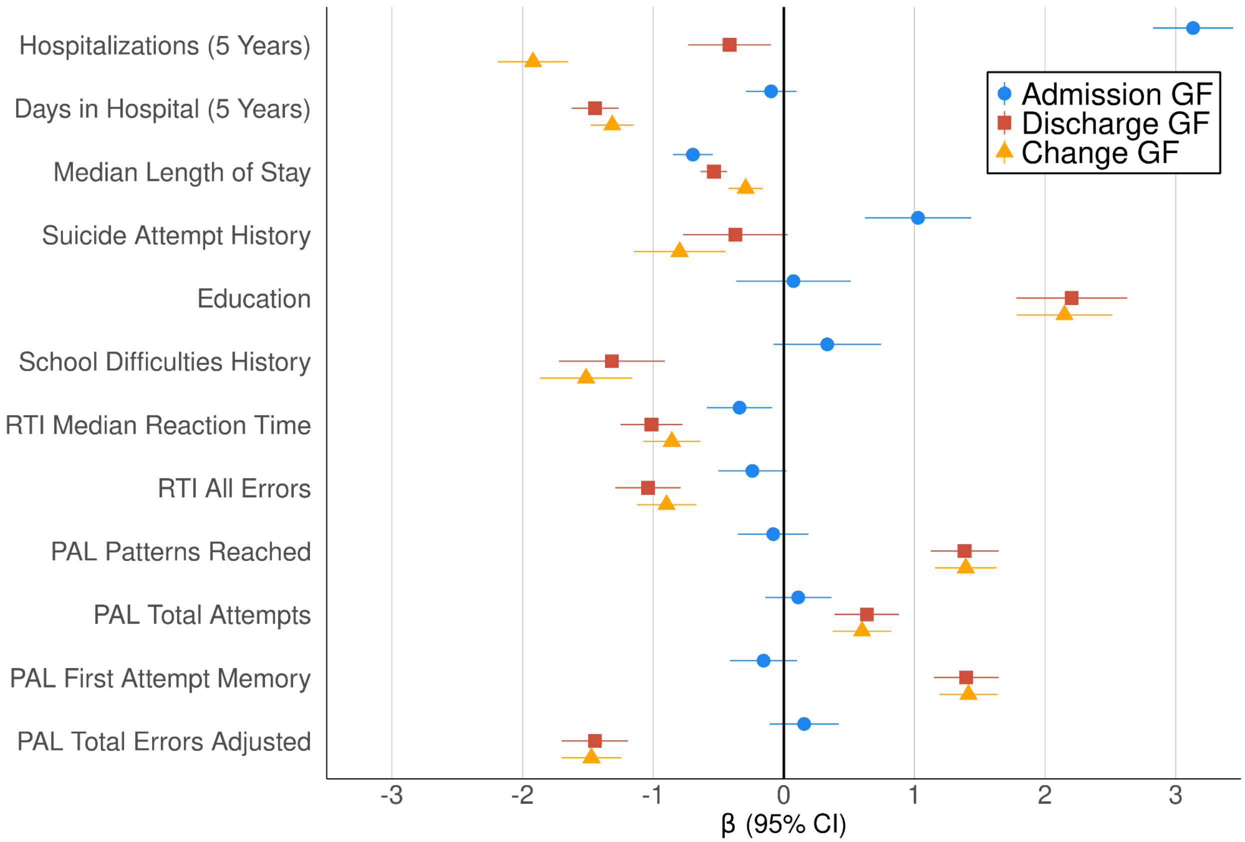

GF: global functioning. Change GF = Discharge GF adjusted for Admission GF. RTI: Reaction Time. PAL: Paired Associates Learning. 5 Years: these variables were calculated for psychiatric hospitalizations within the initial 5 years following a participant's first admission (Supplement). RTI and PAL were assessed at study entry (between November 2015 and December 2018). Bars depict 95% CIs

Figure 3. Schizophrenia and EA PGS Predict Global Functioning Across Hospitalization

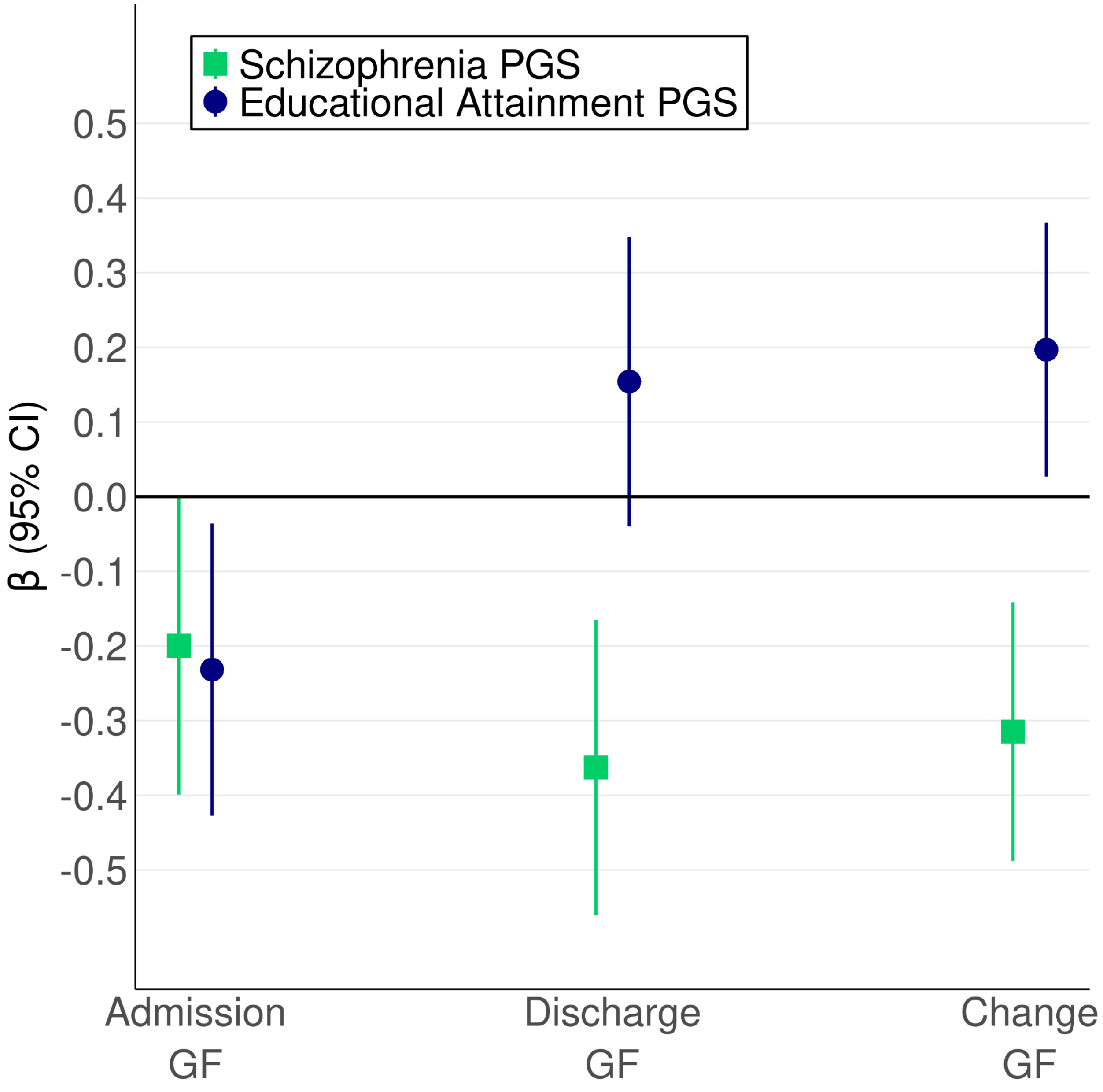

PGS: polygenic score. GF: global functioning. Change GF: Discharge GF adjusted for Admission GF. Bars depict 95% CIs.
